# Thermoring basis for the TRPV3 bio-thermometer

**DOI:** 10.1038/s41598-023-47100-0

**Published:** 2023-12-07

**Authors:** Guangyu Wang

**Affiliations:** 1 Department of Physiology and Membrane Biology, University of California School of Medicine, Davis, CA 95616 USA; 2 Department of Drug Research and Development, Institute of Biophysical Medico-Chemistry, Reno, NV 89523 USA

**Keywords:** Biochemistry, Biophysics, Computational biology and bioinformatics, Physiology, Neurology

## Abstract

The thermosensitive transient receptor potential (TRP) channels are well-known as bio-thermometers with specific temperature thresholds and sensitivity. However, their precise structural origins are still mysterious. Here, graph theory was used to test how the temperature-dependent non-covalent interactions as identified in the 3D structures of thermo-gated TRPV3 could form a systematic fluidic grid-like mesh network with the constrained thermo-rings from the biggest grids to the smallest ones as necessary structural motifs for the variable temperature thresholds and sensitivity. The results showed that the heat-evoked melting of the biggest grids may control the specific temperature thresholds to initiate channel gating while the smaller grids may be required to secure heat efficacy. Together, all the grids along the lipid-dependent minimal gating pathway may be necessary to change with molar heat capacity for the specific temperature sensitivity. Therefore, this graph theory-based grid thermodynamic model may provide an extensive structural basis for the thermo-gated TRP channels.

The thermosensitive transient receptor potential (TRP) channels are well known as biothermometers involving TRPV (vanilloid), TRPM (melastatin), TRPC (canonical), and TRPA (ankyrin). Their temperature thresholds (T_th_) for activation range from noxious cold, cold, warm to noxious heat. Specifically, TRPV1 (> 42 °C), TRPV2 (> 52 °C), TRPV3 (> 32–39 °C), TRPV4 (> 25–35 °C), TRPM2, TRPM3, TRPM4, and TRPM5 are involved in warm to hot sensation. In contrast, TRPA1 (< 17 °C) or TRPM8 (< 20–28 °C) and TRPC5 (< 25–37 °C) are sensitive to cold and cool temperatures. When compared to non-temperature-sensitive ones, they also have a high temperature sensitivity Q_10_ (the ratio of rates or open probabilities (P_o_) of an ion channel measured 10 °C apart)^1–16^. However, the precise structural origins of the specific temperature thresholds and sensitivities are still unknown.

Of special interest, TRPV3, which is mainly expressed in skin keratinocytes and oral and nasal epithelia mediating thermal reception and pain sensation^5,6^, undergoes sensitization together with TRPV2 while TRPV1 and TRPV4 channels desensitize in response to successive heat stimuli^1,7,8,17–19^. Upon initial short heat stimulation within 100 ms, TRPV3 exhibits the high temperature threshold and sensitivity in the noxious temperature range above 50 °C. After that intensive stimulation, it becomes responsive to warm temperatures with the low sensitivity. Further study showed that the insertion of valine at position 412 dramatically eliminates the use-dependent heat sensitization of TRPV3^19^.

Following those findings, the primary cryo-electron microscopy (cryo-EM) structural studies indicated that TRPV3 is a homotetramer. Each monomer has S1-S6 as a transmembrane domain (TMD) and a large intracellular amino- (N-) terminus as an ankyrin repeat domain (ARD). S1-S4 form a voltage-sensor-like domain (VSLD) while S5-S6 and the pore helix and two pore loops are folded as a pore domain. Both the VSLD and the pore domain are swapped via a S4-S5 linker. The TRP helices, which are almost parallel to the membrane, interact with both the skirt ARD and the TMD. Several lipid sites were also found in their interfaces^20^. The pre-S1 domain, together with the carboxyl- (C-) terminal loop domain, couples the TMD with the ARD. The residues ^638^GLGD^641^ in the P-loop-extended region line the selectivity filter to permeate partially hydrated Na^+^, K^+^ or Ca^2+^ ions but not to function as an upper gate. In contrast, the narrowest pore constriction around M677 on S6 may act as a lower gate for channel closure^20,21^. Although the state- and redox-dependent cryo-EM structures of mouse TRPV3 (mTRPV3) with or without the Y564A mutation at different temperatures are available^22–24^, the specific structural motifs responsible for the use-dependent temperature threshold and sensitivity have not been pinpointed precisely.

On the other hand, following the observations that a nucleic acid hairpin can function as a thermo-ring with the number of H-bonds in the stem and the loop length to regulate the melting temperature threshold (T_m_)^25–27^, a graph theory-based grid thermodynamic model has recently been developed to describe protein as a systematic fluidic grid-like noncovalent interaction mesh network along a single polypeptide chain. Further, the T_m_ of each constrained grid and the grid-based systematic thermal instability (T_i_) have been defined and calculated and compared with relevant experimental values. In this way, the theoretical and experimental match allows the thermo-rings from the biggest grid to the smallest one to be identified in turn as the necessary structural motifs for the thermal stability and activity of globular proteins such as two classes of fructose aldolases from psychrophilic to mesophilic and hyperthermophilic^28–30^. More importantly, the thermoring-based heat activation switches in membrane protein TRPV1 have also been identified for the matched temperature thresholds, and all the thermorings of various sizes from the biggest grids to smaller ones along the phosphatidylinositol (PI)-dependent gating pathway have been shown as required for the matched high temperature sensitivity^31^. In this regard, it is necessary to test if TRPV3 should also use such a series of thermo-rings as necessary structural motifs to achieve the use-dependent thermal sensitization.

Here, graph theory was used to examine this hypothesis by carefully decrypting each constrained grid in the grid-like non-covalently interacting mesh networks as identified in the cryo-EM structures of mTRPV3 at 4 °C and 42 °C^23,24^. Once the biggest grid was identified, the calculated T_m_ was compared with the experimental threshold. Further, the grid-based systematic thermal instability (T_i_) was also calculated as important energetic references to identify different gating states for the use-dependent heat sensitization. Finally, the systematic structural thermo-sensitivity (Ω_10_), which has been defined as a heat-evoked change in the total chemical potential of all the grids upon a change in the total molar enthalpy included in non-covalent interactions along the same gating pathway of one subunit between two gating states within 10 °C apart^31^, was also calculated and compared with the experimental Q_10_. Once all the three lines of calculated parameters were found close to the experimental ones of some redox- and lipid-dependent gating states, a closed and reduced state, a sensitized but oxidized state, and an open and oxidized state were identified with a reasonable energetic rearrangement for the use-dependent heat sensitization of TRPV3. The relevant thermodynamic parameters including changes in molar enthalpy, entropy and heat capacity upon channel opening were also calculated and compared and discussed.

## Results

### Definition of the necessary minimal gating pathway for heat-sensing

Previous studies demonstrated that the pre-S1 segment 358–434 plays a critical role in mediating the temperature threshold (T_th_) and sensitivity Q_10_^32^, and the insertion of valine at position 412 is enough to remove the use-dependent heat sensitization of TRPV3^19^. It was further found that the release of the phosphatidylcholine (PC) lipid from the vanilloid site is required for heat-evoked TRPV3 opening^22,24^. Thus, the PC pocket is the pivotal active site, involving the VSLD and the TRP domain^23,24^. The primary scanning of the recent 3D structures of mTRPV3 with or without a C612-C619 disulfide bond in the outer pore indicated that E704 in the C-terminus of the TRP domain bridged with T397 in the distal N-terminus of the pre-S1 domain^23,24^. In this regard, like the segment from D388 in the pre-S1 domain to K710 in the TRP domain of rat TRPV1 (rTRPV1)^31^, the equivalent segment from D396 in the pre-S1 domain to K705 in the TRP domain should be at least included as the necessary PC-dependent minimal gating pathway for the matched temperature threshold and sensitivity^23,24^.

### Identification of a heat switch in oxidized mTRPV3 for channel opening with low T_m_ and Ω_10_

Oxidized mTRPV3 with the disulfide bond between C612 and C619 in the outer pore has been reported to open from a PC-bound closed state at a lower threshold 42 °C after repeated heat sensitization from 25 °C to 40 °C^24^. Therefore, it is exciting to test if oxidation is responsible for the low experimental temperature threshold and sensitivity^19^.

**Figure 1:**
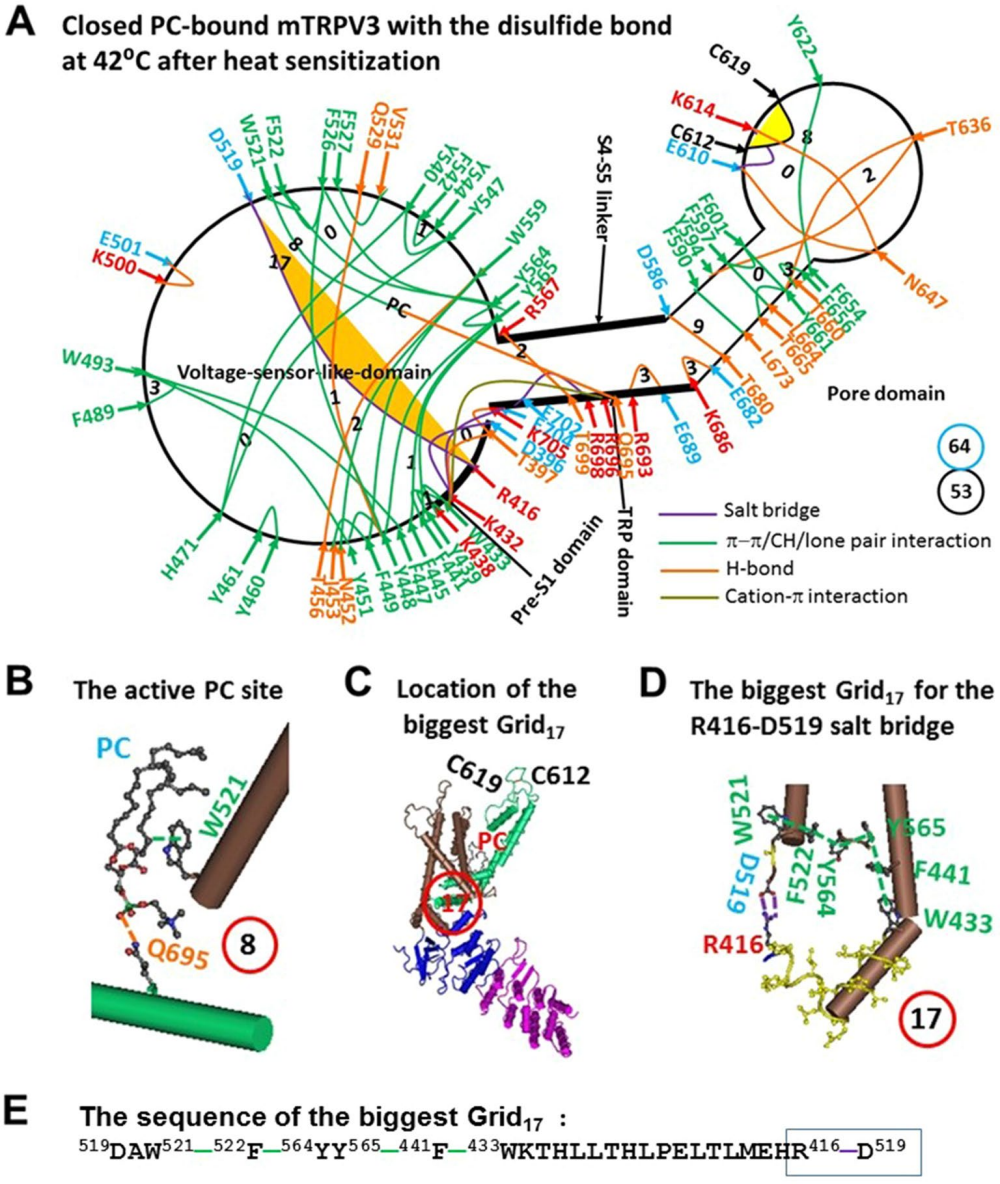
The grid-like non-covalently interacting mesh network along the PC-dependent minimal gating pathway of PC-bound oxidized mTRPV3 in the sensitized state at 42 °C after heat sensitization. (**A**) The topological grids in the systemic fluidic grid-like noncovalent interaction mesh network. The cryo-EM structure of one subunit in sensitized and oxidized mTRPV3 with PC bound at the vanilloid site in cNW11 at 42 °C (PDB ID, 7MIN) was used for the model. The pore domain, the S4-S5 linker, the TRP domain, the VSLD and the pre-S1 domain are indicated in black. Salt bridges, π interactions, and H-bonds between pairing amino acid side chains along the gating pathway from D396 to K705 are marked in purple, green, and orange, respectively. The disulfide bond between C612 and C619 was highlighted. The grid sizes required to control the relevant non-covalent interactions were calculated with graph theory and labeled in black. The R416-D519 salt bridge in the biggest Grid_17_ was highlighted. The total grid sizes and grid size-controlled non-covalent interactions along the PC-dependent minimal gating pathway from D396 to K705 are shown in the cyan and black circles, respectively. (**B**) The structure of the active PC lipid site. (**C**) The location of the biggest Grid_17_ is marked in a red circle. (**D**) The structure of the biggest Grid_17_ with a 17-residue size in the VSLD/pre S1 interface to control the R416-D519 salt bridge. (**E**) The sequence of the biggest Gird_17_ to control the R416-D519 salt bridge in a blue box.

Along the defined necessary PC-dependent minimal gating pathway from D396 to K705 in the closed state, non-covalent interactions between amino acid side chains were diversified. They included five salt bridges, fourteen H-bonds and thirty-four π interactions (Fig. 1A, Table S1). Of special interest, the PC lipid was sandwiched by W521 in the VSLD and Q695 in the TRP domain via an H-bond and a CH-π interaction (Fig. 1B). When all the noncovalent interactions formed a systematic fluidic grid-like mesh network, the total non-covalent interactions and grid sizes were 53 and 64, respectively (Fig. 1A). Thus, the grid-based systemic thermal instability (T_i_) was about 1.21 (Table 1). Despite several smallest grids with a zero-residue size, the biggest Grid_17_ with a 17-residue size was outstanding in the VSLD/pre-S1 interface and near the PC-lipid to control the D519-R416 salt bridge (Fig. 1B–D). It started with D519 and went through W521, F522, Y564, Y565, F441, W433 and ended with R416 (Fig. 1E). When two equivalent H-bonds sealed the grid, the predicted T_m_ was about 40 °C (Table 1), which was close to the measured T_m_ 42 °C^24^.

**Table 1:** The grid thermodynamic model-based new parameters of the mTRPV3 bio-thermometer along the PC-dependent minimal gating pathway or the extended.

Construct	Wild-type mTRPV3						
PDB ID	6LGP		7MIO		7MIN		
Lipid PC at the vanilloid site	Bound		Free		Bound		
Redox state	Reduced		Oxidized		Oxidized		
Lipid environment	MSP2N2		cNW11		cNW11		
Sampling temperature, °C	**4**		**42**		**42**		
Gating state	Closed		Open		Sensitized		
# of the biggest grid	Grid_11_		Grid_9_		Grid_17_		
Biggest grid size (S_max_)	11		9		17		
Equivalent H-bonds controlled by S_max_	2.0		2.5		2.0		
Channel gating pathway	396–705	377–742	396–705	377–742	396–705		377–742
Total non-covalent interactions	51	60	49	61	53		66
Total grid sizes, a.a	96	117	59	76	64		88
Calculated T_m_, °C	**52**		**61**		**40**		
Measured T_m_, °C					**42**		
Measured T_th_, °C	**52**		**62**		**32–39**		
T_1/2_, K	**329.5**	**329.5**			**323.5**		**323.5**
∆G, kcal/mol	** − 18.5**	** − 20.5**			** − 2.5**		** − 6**
∆H_1/2_, kcal/mol	**4**	** − 2**			**8**		**10**
∆S_1/2_, cal/mol-K	**12.1**	** − 6.07**			**24.7**		**30.9**
∆Cp, kcal/mol-K	**8.68**	**9.61**			**0.762**		**1.83**
Systemic thermal instability (T_i_)	**1.88**	**1.95**	**1.20**	**1.25**	**1.21**		**1.33**
Calculated Ω_10, min_ at E_min_ = 0.5 kcal/mol	10.1	9.78			1.27		3.28
Calculated Ω_10, mean_ at E_mean_ = 1.0 kcal/mol	**20.8**	**19.3**			**2.69**		**6.92**
Calculated Ω_10, max_ at E_max_ = 3.0 kcal/mol	65.2	56.6			8.81		22.7
Measured Q_10,_	**20.6**				**2.32**		
Ref. for measured T_th_ or Q_10_	^19^		^19,34^		^19,24^		

The comparative parameters are highlighted in bold. The measured T_th_ values were derived from the previous experimental data by using a traditional intersection between a silent line and a maximal active line^19^.

**Figure 2:**
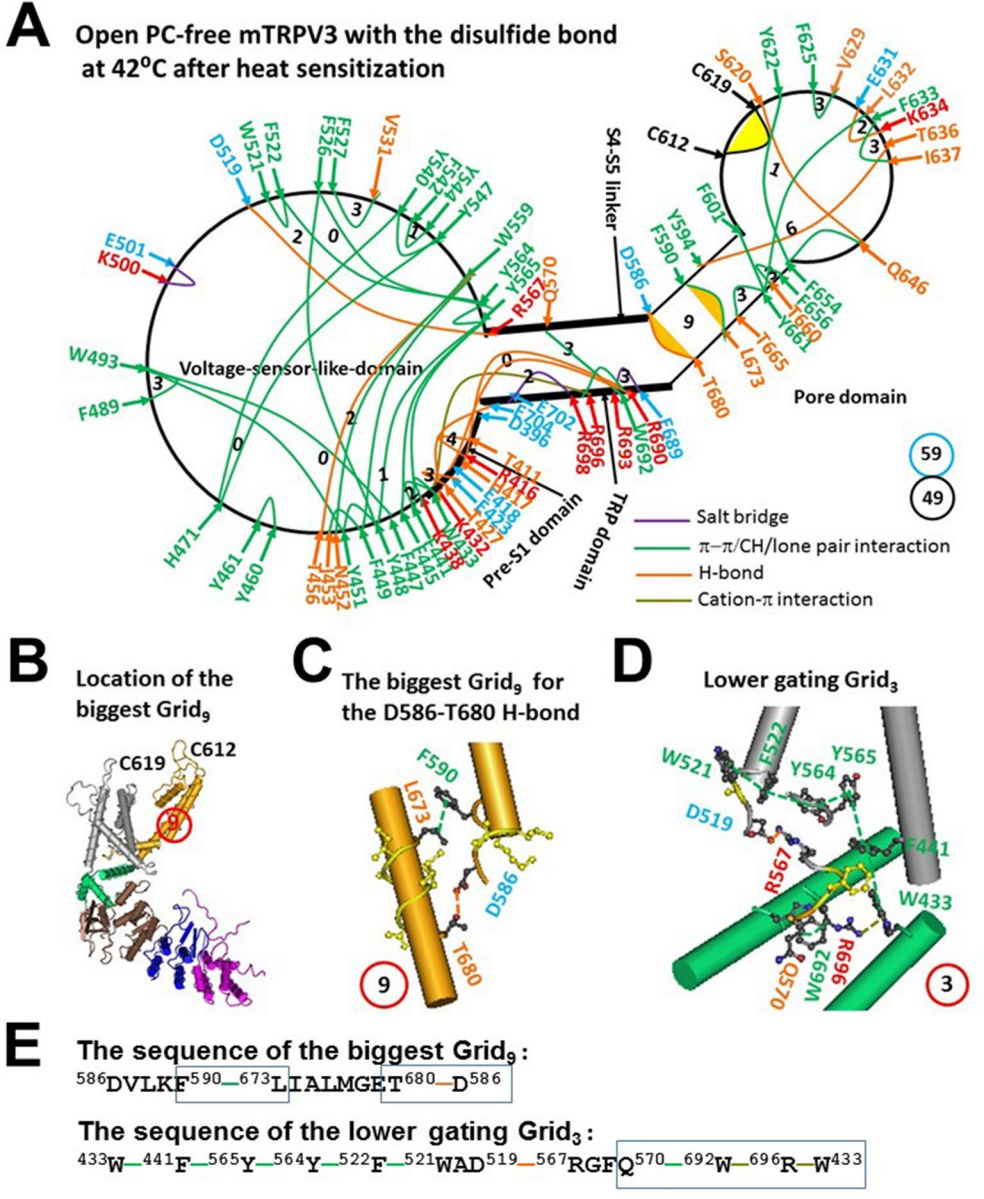
The grid-like non-covalently interacting mesh network along the PC-dependent minimal gating pathway of PC-free oxidized mTRPV3 in the open state at 42 °C after heat sensitization. (**A**) The topological grids in the systemic fluidic grid-like noncovalent interaction mesh network. The cryo-EM structure of one subunit in open and oxidized mTRPV3 without PC bound at the vanilloid site in cNW11 at 42 °C (PDB ID, 7MIO) was used for the model. The pore domain, the S4-S5 linker, the TRP domain, the VSLD and the pre-S1 domain are indicated in black. Salt bridges, π interactions, and H-bonds between pairing amino acid side chains along the PC-dependent minimal gating pathway from D396 to K705 are marked in purple, green, and orange, respectively. The disulfide bond between C612 and C619 was highlighted. The grid sizes required to control the relevant non-covalent interactions were calculated with graph theory and labeled in black. The D586-T680 H-bond and the F590-L673 π interaction in the biggest Grid_9_ were highlighted. The total grid sizes and grid size-controlled non-covalent interactions along the PC-dependent minimal gating pathway are shown in the cyan and black circles, respectively. (**B**) The location of the biggest Grid_9_ is marked in a red circle. (**C**) The structure of the biggest Grid_9_ with a 9-residue size in the S5/S6 interface to control the D586-T680 and F590-L673 bridges. (**D**) The structure of the putative smaller Grid_3_ with a 3-residue size for the lower gate. (**E**) The sequences of gating Grid_9_ and Grid_3_ to control the D586-T680 and F590-L673 bridges and a group of crtitical cation-π interactions in the blue boxes, respectively.

In agreement with the predicted T_m_ 40 °C, the R416-D519 salt bridge in the biggest Grid_17_ of oxidized mTRPV3 was melt at 42 °C for channel opening along with the release of PC from nearby W521 and Q695 (Figs. 1A and 2A, Tables S1 and S2)^24^. As a result, the new biggest Grid_9_ with a 9-residue size was created in the S5/S6 interface, which may be required for channel opening (Fig. 2B,C). When 2.5 equivalent H-bonds sealed Grid_9_ via the shortest path from D586 to F590 and L673 and T680 and back to D586 (Fig. 2C,E), the calculated T_m_ was about 61 °C (Table 1). Since Grid_9_ was conserved in both closed and open states of oxidized mTRPV3 (Figs. 1A and 2A), it may act as a thermostable anchor to secure channel activity below 61 °C. Meanwhile, a smaller Grid_3_ in the pre-S1/VSLD/S4-S5 linker/TRP/pre-S1 interfaces may be required to stimulate the lower gate of the channel. It had a 3-residue size to control the Q570-W692-R696-W433 π interaction chain via the shortest path from W433 to F441, Y565, Y564, F522, W521, D519, R567, Q570, W692, R696, and back to W433 (Fig. 2D,E). In agreement with this proposal, it has been shown that the R567K mutant or the nearby G573S mutant promotes the heat activation but decreases the Q_10_ value^33^.

On the other hand, the total non-covalent interactions and grid sizes along the PC-dependent minimal gating pathway from D396 to K705 decreased from 53 and 64 to 49 and 59, respectively (Figs. 1A and 2A, Table 1). Despite such a decrease, the systemic thermal instability (T_i_) value was still kept around 1.20 (Table 1). Of special note, the systematic structural thermo-sensitivity Ω_10_ was in a range from 1.27 to 8.81 and with a mean value 2.69, which was close to the experimental Q_10_ of 2.32 (Table 1)^19^. Therefore, even if the PC lipid at the corresponding vanilloid site was not released by the Y564A mutation^22^, the presence of the C612-C619 disulfide bond in the outer pore was still adequate for mTRPV3 to open with both low T_m_ and Ω_10_ to match the measured T_th_ and Q_10_ in response to the second heat stimulation (Table 1)^19^. In that regard, it is attractive to test if the disruption of the C612-C619 disulfide bond can increase both T_m_ and Ω_10_ values upon channel opening from reduced mTRPV3 to meet the requirement of the higher T_th_ (> 50 °C) and Ω_10_ (20.6)^19^.

### Identification of another heat switch in reduced mTRPV3 for channel opening with high T_m_ and Ω_10_

**Figure 3:**
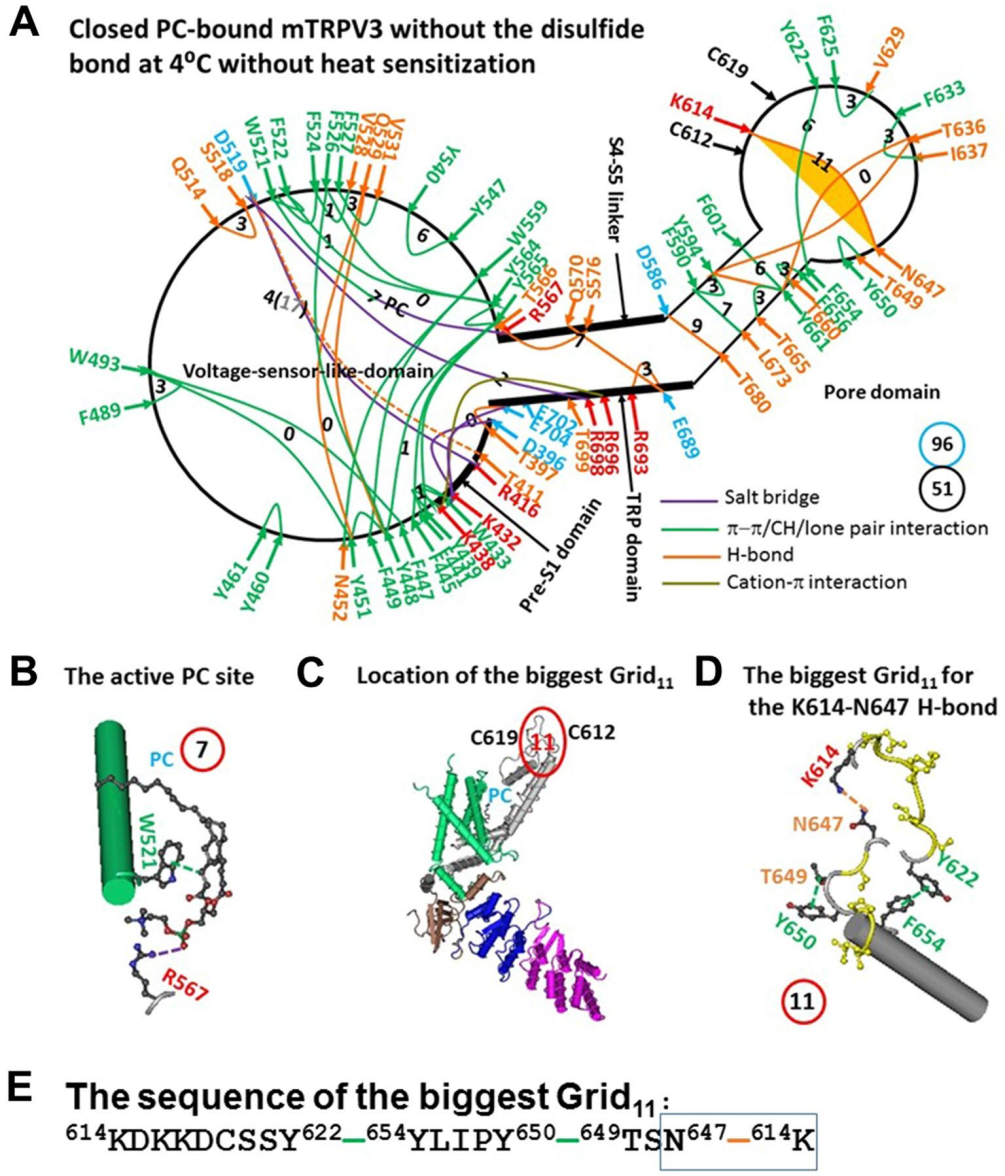
The grid-like non-covalently interacting mesh network along the PC-dependent minimal gating pathway of PC-bound reduced mTRPV3 in the closed state at 4 °C without heat sensitization. (**A**) The topological grids in the systemic fluidic grid-like noncovalent interaction mesh network. The cryo-EM structure of one subunit in reduced and closed mTRPV3 with PC bound at the vanilloid site in MSP2N at 4 °C (PDB ID, 6LGP) was used for the model. The pore domain, the S4-S5 linker, the TRP domain, the VSLD and the pre-S1 domain are indicated in black. Salt bridges, π interactions, and H-bonds between pairing amino acid side chains along the PC-dependent minimal gating pathway from D396 to K705 are marked in purple, green, and orange, respectively. The grid sizes required to control the relevant non-covalent interactions were calculated with graph theory and labeled in black. The K614-N647 H-bond in the biggest Grid_11_ was highlighted. The total grid sizes and grid size-controlled non-covalent interactions along the PC-dependent minimal gating pathway are shown in the cyan and black circles, respectively. The flexible T411-D519 H-bond, which is marked in a dashed line, may be disrupted by the insertion of valine or serine at position 412. (**B**) The structure of the active PC site. (**C**) The location of the biggest Grid_11_ is marked in a red circle. (**D**) The structure of the biggest Grid_11_ with an 11-atom size at the PC site to control the K614-N647 H-bond. (**E**) The sequence of the biggest Grid_11_ to control the K614-N647 H-bond in a blue box.

When the C612-C619 disulfide in closed mTRPV3 was broken, the global conformational wave from the pore domain to the VSLD allowed some different noncovalent interactions to form the distinct systematic fluidic grid-like non-covalent interaction mesh network at 4 °C (Figs. 1A and 3A, Tables S1 and S3)^23^. As a consequence, the PC lipid was linked by W521 and R567 in the VSLD (Fig. 3B). However, when the T411-D519 H-bond and the R416-D519 salt bridge near the PC site generated a smaller Grid_4_ to control both bridges via the shortest path from T411 to R416, D519 and back to T411 (Fig. 3A), the calculated T_m_ of Grid_4_ was up to 71 °C. In this case, the PC lipid could not be released by the disruption of the nearby R416-D519 salt bridge for channel opening at lower temperature 32–39 °C^19^. On the other hand, the biggest Grid_11_ was actually born in the outer pore (Fig. 3C). When two equivalent H-bonds governed the K614-N647 bridge via the shortest path from K614 to Y622, Y654, Y650, T649, N647 and back to K614 (Fig. 3D–E), the predicted melting temperature threshold (T_m_) was about 52 °C (Table 1), which was the same as the initial experimental T_th_ 52 °C for TRPV3 opening^19^. What is more, when compared with oxidized mTRPV3 in both closed and open states, only 2 H-bonds and 27 π interactions were conserved, and five new salt bridges and 11 new H-bonds and 6 new π interactions were added (Figs. 1A, 2A, 3A, Tables S1, S2, S3). As the total non-covalent interactions and grid sizes along the PC-dependent minimal gating pathway from D396 to K705 were 51 and 96, respectively (Fig. 3A), the systemic thermal instability (T_i_) was increased from 1.21 to 1.88 (Table 1). When the same open state as shown in the oxidized and PC-free mTRPV3 was employed as a control (Fig. 2A), the melting of the K614-N647 H-bond in the biggest Grid_11_ would produce the calculated Ω_10_ in a range from 10.1 to 65.2 and with a mean value 20.8, which approximated to the experimental Q_10_ of 20.6 (Table 1)^19^. Hence, the initial high T_th_ and Q_10_ of mTRPV3 upon the brief heat stimulation may result from the gating transition from the reduced and closed state to the open and oxidized one.

### The role of interactions between distal N- and C- termini in mTRPV3

Previous chimera study indicated that the replacement of the cytoplasmic inter-subunit interface (N251-E257) of human TRPV3 (hTRPV3) with the homologous residues of rTRPV1 prohibits the use-dependent sensitization^34^. As the engineered interfacial disulfide bond between F259C and V385C’ locks the channel in the open state, it was proposed that the ARD-β sheet inter-subunit interface is a critical element of the temperature-sensing molecular machinery of TRPV3^34^. The subsequent cryo-RM structure of mTRPV3 further demonstrated that the intracellular skirt of TRPV3 rotates by ~ 8° and moves towards the transmembrane domain in response to the heat stimulus^24^. Thereby, it is necessary to evaluate the role of the noncovalent interactions between N- and C- terminal domains in heat-evoked channel gating of mTRPV3.

**Figure 4:**
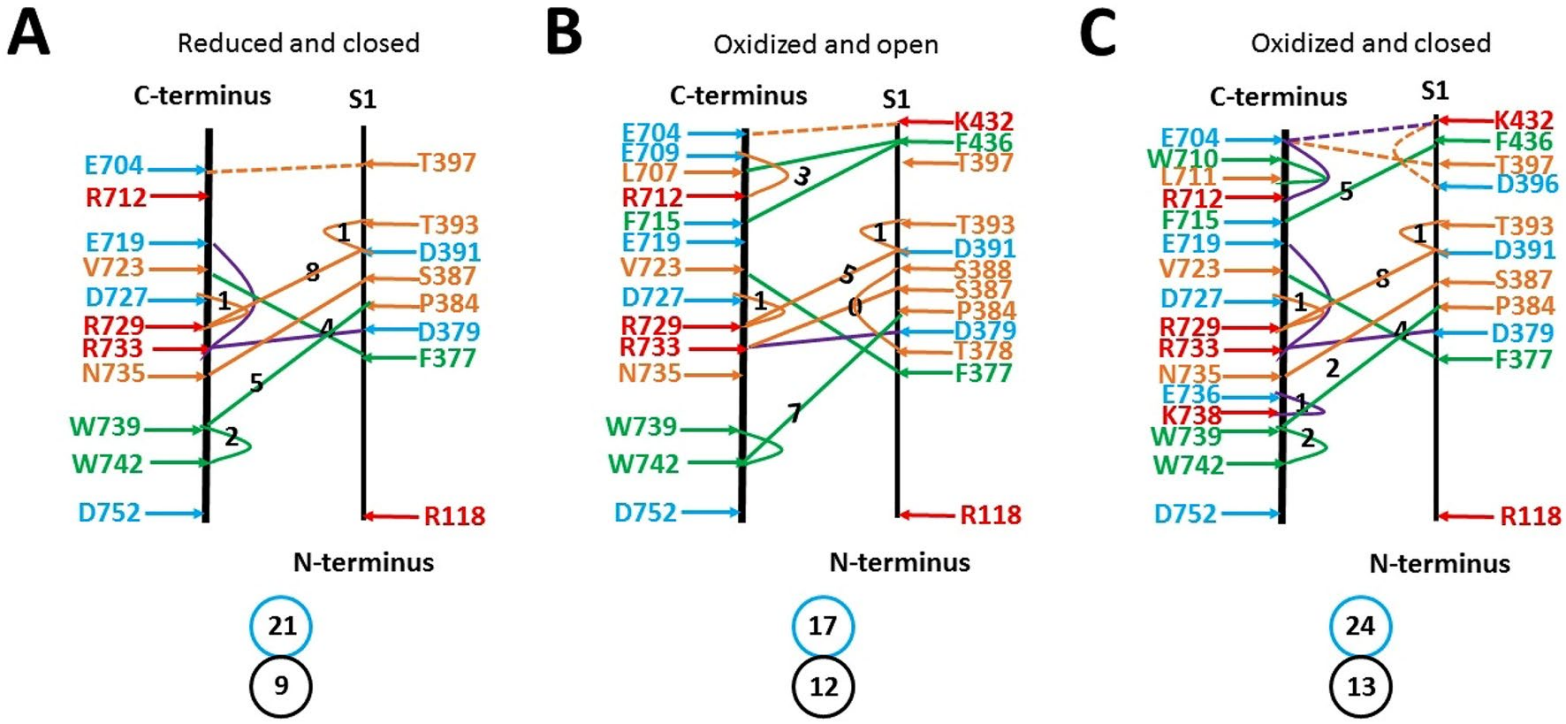
The grid-like non-covalently interacting mesh network between N- and C-terminal domains of mTRPV3 beyond the PC-dependent minimal gating pathway. (**A**) Reduced and closed mTRPV3 with PC-bound at 4 °C (PDB ID, 6LGP). (**B**) Oxidized and open mTRPV3 without PC-bound at 42 °C (PDB ID, 7MIO). (**C**) Oxidized and closed mTRPV3 with PC-bound at 42 °C (PDB ID, 7MIN). The N- and C-terminal domains are indicated in black along with S1. Salt bridges, π interactions, and H-bonds between the N- and C-terminal domains are marked in purple, green, and orange, respectively. The grid sizes required to control the relevant non-covalent interactions were calculated with graph theory and labeled in black. The dashed lines were non-covalent interactions along the PC-dependent minimal gating pathway from D396 to K705. The total grid sizes and grid size-controlled non-covalent interactions between N- and C- termini are shown in the cyan and black circles, respectively.

When the systematic fluidic grid-like noncovalent interaction mesh networks extended to F377 in the N-terminus and W742 in the C-terminus, the sizes of the additional biggest grids were 8 and 7 in the closed and open states, respectively (Fig. 4). Since no biggest grid in this interface had the size larger than that along the PC-dependent minimal gating pathway from D396 to K705 in either gating state (Table 1), the heat starter was not located in the interface between N- and C-terminal domains in either gating state.

On the other hand, the total additional noncovalent interactions and grid sizes were 9 and 21 in the reduced and closed state, 12 and 17 in the oxidized and open state, and 13 and 24 in the oxidized and closed state, respectively (Fig. 4). When these values were included, the systematic thermal instability (T_i_) increased from 1.88 to 1.95 for reduced and closed mTRPV3, from 1.20 to 1.25 for oxidized and open mTRPV3, and from 1.21 to 1.33 for oxidized and closed mTRPV3 (Table 1). Meanwhile, the mean structural thermosensitivity (Ω_10_) changed from 20.8 to 19.3 for reduced mTRPV3 but from 2.69 to 6.92 for oxidized one (Table 1). Accordingly, the PC-dependent minimal gating pathway from D396 in the pre-S1 domain to K705 in the TRP domain was enough to bring about the matched temperature thresholds and sensitivity with the low systematic thermal instability (T_i_). Further extension of the gating pathway may be unnecessary.

Notably, several noncovalent interactions in the interface between N- and C-termini were conserved in three above gating states. They included F377-V723 and W739-W742 π interactions, a D379-R733 salt bridge, and D727-R729-D391-T393 H-bonds (Fig. 4). Thus, these gating-independent noncovalent interactions may facilitate the assembly of the channel. Like the C612-C619 disulfide bond in the outer pore, any perturbation near the ARD-β sheet inter-subunit interface may allosterically allow the biggest Grid_17_ in the VSLD/pre-S1 interface to control a lower threshold to activate TRPV3 (Fig. 1A)^34^.

### Evaluation of molar heat capacity for channel opening from the closed states of oxidized and reduced mTRPV3

When oxidized mTRPV3 was gated from the closed state at 40 °C to the open state until the maximal activity temperature around 61 °C, the total noncovalent interactions and grid sizes along the PC-dependent minimal gating pathway of one subunit decreased from 53 and 64 to 49 and 59, respectively (Table 1). Assuming the apparent channel open probability (P_o_) was 0.5 at T_1/2_ of 50.5 °C (323.5 K), which was close to the experimental value^19^, the change in molar enthalpy at 323.5 K (ΔH_1/2_) upon the total broken non-covalent interactions would be 8 kcal/mol (Table 1). Thus, the change in molar entropy at 323.5 K (ΔS_1/2_) was 24.7 cal/mol-K (Table 1). Assuming mTRPV3 was allosterically open at 61 °C, the change in molar Gibbs free energy (ΔG) would be − 2.5 kcal/mol (Table 1). Based on the modified Gibbs–Helmholtz equation, the change in molar heat capacity (ΔC_p_) was about 0.762 kcal/mol-K for one channel (Table 1). When the gating pathway was extended beyond the PC-dependent minimal gating pathway, the ΔC_p_ value increased to 1.83 kcal/mol-K (Table 1).

In contrast, for channel gating from the closed and reduced state at 52 °C to the open and oxidized one until the maximal activity temperature around 61 °C, the total noncovalent interactions and grid sizes along the PC-dependent minimal gating pathway of one subunit decreased from 51 and 96 to 49 and 59, respectively (Table 1). If the apparent P_o_ was 0.5 at T_1/2_ of 56.5 °C (329.5 K), which was still near the experimental value^19^, ΔH_1/2_ and ΔS_1/2_ at 329.5 K upon the total broken non-covalent interactions would be 4 kcal/mol and 12.1 cal/mol-K, respectively (Table 1). If mTRPV3 allosterically opened at 61 °C, ΔG would be − 18.5 kcal/mol (Table 1). Using the same modified Gibbs–Helmholtz equation, ΔC_p_ was calculated as large as 8.68 kcal/mol-K for one channel (Table 1). The value increased to 9.61 kcal/mol-K along with the extended gating pathway from F377 to W742 (Table 1). Clearly, oxidized mTRPV3 may favor the use-dependent sensitization with less ΔC_p_.

## Discussion

The TRPV3 biothermometer is characterized as the use-dependent heat sensitization. Although several cryo-EM structures of mTRPV3 are available in different gating and redox states and at various temperatures, the specific structural motifs responsible for this characterization are still unresolved precisely. This computational study first demonstrated that the calculated melting temperature threshold (T_m_) of the biggest grid along the PC-dependent minimal gating pathway in mTRPV3 was comparable to not only the structural T_m_ but also the functional activation threshold T_th_ of mTRPV3. It further confirmed that the functional thermo-sensitivity Q_10_ was also comparable to the grid-based structural thermo-sensitivity Ω_10_, and related to the change in molar heat capacity along the PC-dependent minimal gating pathway. Finally, the grid-based systematic thermal instability values of mTRPV3 in different redox- and lipid-dependent gating states were also compared with each other to establish the energetic relationship of different gating states. Taken as a whole, three gating states were completely identified to account for the use-dependent heat sensitization of TRPV3.

First, it was further confirmed that the biggest grid may employ its size and strength to determine the melting temperature threshold (T_m_) of TRPV3. At a given salt concentration (150 mM NaCl), for oxidized and sensitized mTRPV3 in the closed state, when two equivalent H-bonds sealed the biggest Grid_17_ to control the R416-D519 salt bridge in the interface between the S2-S3 linker and the pre-S1 domain (Fig. 1A,C–E), it had a calculated T_m_ 40 °C (Table 1). Although previous molecular dynamics (MD) simulations of closed mTRPV3 revealed the highly flexible S2-S3 linker at 300 K^34^, the subsequent structural data of mTRPV3 showed that the R416-D519 salt bridge in the biggest Grid_17_ of oxidized and closed mTRPV3 was factually maintained from 4 °C to 42 °C no matter whether the channel is reconstituted in MSP2N2 or cNW11^24^. Furthermore, only in the open state was such a key salt bridge melt at 42 °C (Figs. 1A and 3A, Table 1)^24^. Thereafter, the biggest Grid_17_, even if identified not at 42 °C but at 4 °C, was not affected by the MD simulations. In other words, the heat activation switch with a specific threshold could also be defined from the high-resolution cryo-EM structure of the protein at a lower temperature. In this regard, the biggest Grid_11_ as found in the outer pore of reduced mTRPV3 at 4 °C could govern the K614-N647 H-bond for the matched activation threshold of 52 °C (Fig. 3A,C–E, Table 1).

Second, if the functional temperature threshold (T_th_) for activation of mTRPV3 is controlled by the melting temperature threshold (T_m_) of the biggest grid along the PC-dependent minimal gating pathway, the calculated T_m_ should be comparable to the measured threshold T_th_. In accordance with this prediction, reduced mTRPV3 in the closed state had a T_th_ around 52 °C which was the same as the calculated T_m_ 52 °C of the biggest Grid_11_ in the outer pore (Fig. 3A,C–E, Table 1)^19^. Once oxidized in the pore, mTRPV3 had a low calculated T_m_ of 40 °C (Table 1). Because of the flexible S2-S3 linker^34^, when the R416-D519 salt bridge changed the strength from 0.5 to 2.0 equivalent H-bonds in response to the tunable distance, the activation thresholds may be allowed to range from 25 to 40 °C^5,6,19,29^. Hence, the activation threshold T_th_ may be governed by the melting of the biggest grid via the adjustable R416-D519 salt bridge along the PC-dependent minimal gating pathway. In agreement with this notion, the similar E406-K504 salt bridge in the interface between the pre-S1 domain and the S2-S3 linker of hTRPV1 is also identified in the biggest Grid_14_ for the matched T_th_ of 41 °C^31^. When the segment ^365^KD^366^ of rTRPV2 is replaced with the equivalent ^405^ET^406^ of rTRPV1, the activation threshold T_th_ decreases from 52 °C to 46 °C^35^. Since the calculated T_m_ value of the biggest Grid_9_ in the open state of oxidized mTRPV3 was about 61 °C (Fig. 2A, Table 1), the maximal activity temperature limit may be around 61 °C^19,34^.

Third, when TRPV3 channel opening from a closed or sensitized state within 10 °C was initially driven by the change in the systematic molar enthalpy as a result of the broken biggest Grid_17_ or Grid_11_, the functional thermo-sensitivity (Q_10_) should be comparable to the calculated systematic structural thermo-sensitivity Ω_10_ because they both factually reflect the change of the total chemical potentials of all the grids upon the alteration of the total molar enthalpy included in the non-covalent interactions between two gating states along the same PC-dependent minimal gating pathway from D396 to K705. In agreement with this proposal, if wild-type mTRPV3 had the same open and oxidized state, the calculated mean Ω_10_ of reduced mTRPV3 would be 20.8, which was close to the measured Q_10_ (20.6) (Table 1)^19^. For oxidized and sensitized mTRPV3, the calculated mean Ω_10_ was 2.69, which was similar to the measured Q_10_ (2.32) (Table 1)^19^. Thereafter, the functional thermo-sensitivity Q_10_ may be governed by the grid-based systematic structural thermo-sensitivity Ω_10_ as defined previously^31^. When the intensity of a non-covalent interaction was in the range from 0.5 to 3 kcal/mol^31^, the resultant Ω_10_ ranges from the minimum to the maximum may be theoretically calculated as 10.1–65.2 for reduced and closed mTRPV3, and 1.27–8.81 for sensitized and oxidized mTRPV3 (Table 1).

It should be noteworthy that even if the change in molar enthalpy (ΔH) of one channel upon the total broken non-covalent interactions was as small as 8 kcal/mol from a closed and reduced state to an oxidized and open one, the huge change in molar heat capacity (ΔC_p_) of 8.68 kcal/mol-K was still enough to absorb more heat as the configurational heat capacity (love) while keeping a small change in molar entropy of open conformations at T_1/2_ (ΔS_1/2_, 12.1 cal/mol). This observation was in agreement with the decrease in the entropy of compact conformations as reflected by the decrease of the systematic thermal instability from 1.88 to 1.20 to stabilize the apparent open state (Table 1). In sharp contrast, when mTRPV3 was oxidized, ΔC_p_ dramatically declined from 8.68 kcal/mol-K to 0.762 kcal/mol-K along with the decrease in the mean Ω_10_ value from 20.8 to 2.69 and the decline in Q_10_ from 20.6 to 2.32 (Table 1). Therefore, the specific temperature sensitivity was closely related to the change in molar heat capacity.

Traditionally speaking, ΔC_p_ along with the thermal unfolding of the protein is linked predominantly with the ordering of water molecules when hydrophobic residues are exposed to the solvent, and thus correlated with changes in solvent accessible surface areas^36^. However, this computational study demonstrated that the high ΔC_p_ effects may be expected for any system made up of a multiplicity of weak noncovalent interactions, of which hydrophobic ones are just a special case.

**Figure 5:**
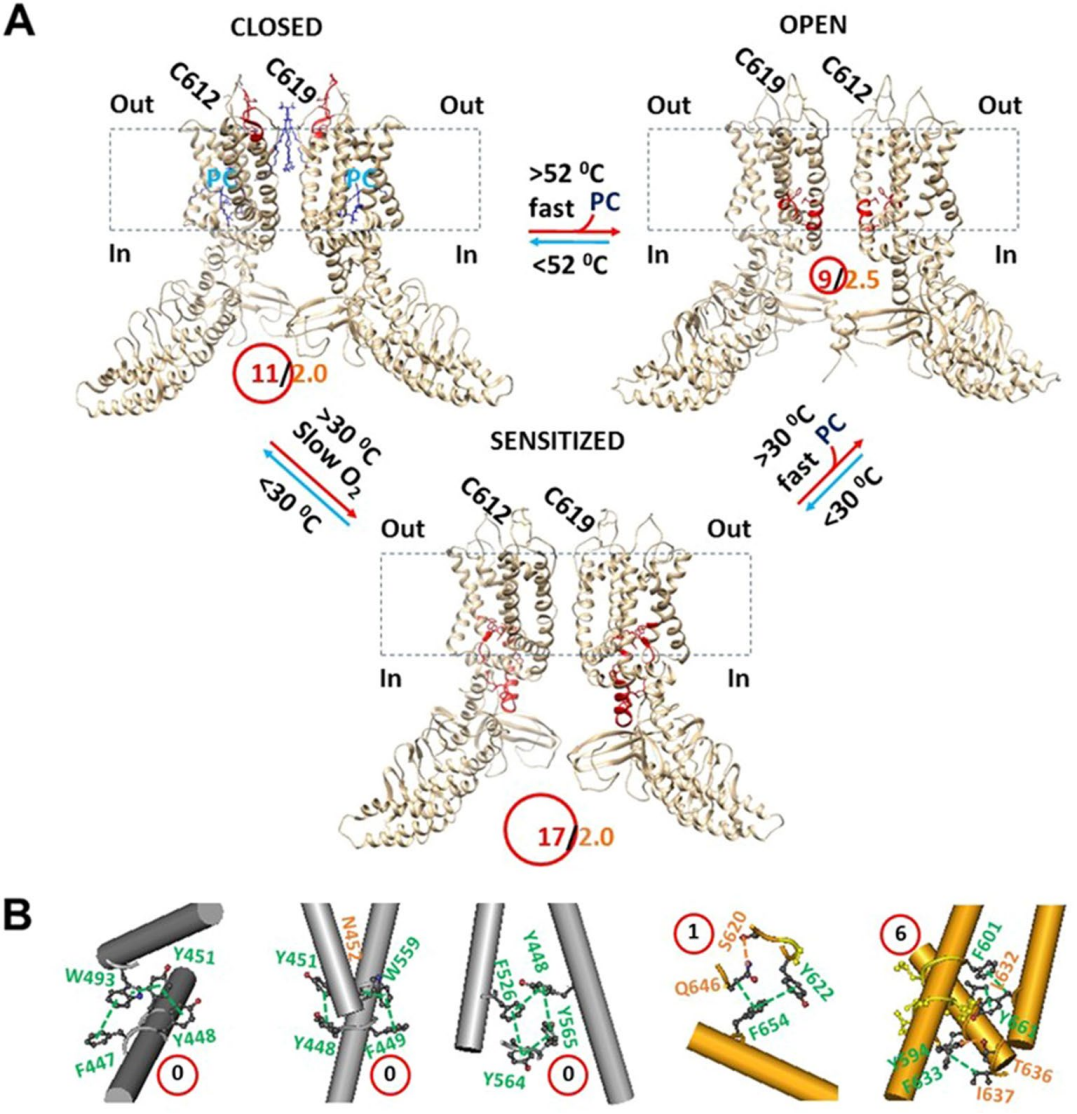
The tentative model for the use-dependent heat sensitization of thermo-gated TRPV3. (**A**) The homo-tetrameric cryo-EM structures of mTRPV3 in the reduced and closed state (PDB ID: 6LGP), the sensitized and oxidized state (PDB ID: 7MIN), and the open and oxidized state (PDB ID: 7MIO) were used for the model. For a convenient view, only two opposite subunits are shown. The dashed rectangle is the membrane area. In the absence of the C612-C619 disulfide bond, reduced mTRPV3 is open with a high Q_10_ (20.6) upon the short heat stimulus above 52 °C to melt the biggest Grid_11_, a thermo-active ring (red), in the outer pore and then to release PC from the active vanilloid site. Meanwhile, C612 is close to C619 enough to form a disulfide bond. When the temperature decreases below 40 °C, oxidized and PC-free mTRPV3 is closed with the biggest Grid_17_, another thermo-active ring (red), in the VSLD to decrease the threshold from 52 °C to 30–40 °C. Upon the second short heat stimulus, the sensitized and oxidized mTRPV3 channel has a Q_10_ as low as 2.32. However, the long and slow warm stimulation above 30 °C can also oxidize mTRPV3 to decrease the threshold from 52 °C to 30 °C in favor of the release of the PC lipid from the vanilloid site for channel opening but with a low Q_10_ (1.66). (**B**) The proposed smaller grids to secure heat efficacy (PDB ID, 7MIO). The grid sizes are shown in the red circles.

Taken together, it is proposed that reduced mTRPV3 may start the first activation above the calculated T_m_ 52 °C upon the fast heat stimulation. Once the channel is opened, it is oxidized to form the C612-C619 disulfide bond so that the functional thermo-sensitivity Q_10_ (20.6) can keep consistent with the calculated Ω_10_ (20.8) (Fig. 5A, Table 1). It is further proposed that when the temperature declines, oxidized but sensitized mTRPV3 may decrease the T_th_ to 30–40 °C and Q_10_ to 2.32 as a result of the formation of the C612-C619 disulfide-bond (Fig. 5A, Table 1)^19^. In this way, the lower threshold 30–40 °C may increase the open probability in response to the same temperature jump from 32 °C to 59 °C so that mTRPV3 activation exhibits the use-dependent sensitization upon successive heat stimuli^19^. In direct line with this proposal, the grid-based systemic thermal instability (T_i_) in the closed state was 1.88 for reduced mTRPV3, and decreased to 1.21 when oxidized by heat sensitization in favor of the use-dependent heat-sensitization during channel opening with a similar T_i_ of 1.20 (Table 1).

On the other hand, when reduced or Cys-less mTRPV3 is exposed to the long and slow heat stimulation^37^, the channel can be activated above a threshold T_th_ 30 °C. Following those observations, several chimeric and mutation studies have been reported to lower down the initial T_th_ from 52 °C to 30 °C and the related Q_10_ upon the short heat stimulus, including the chimeric replacement of the segment N410-D414 in the pre-S1 domain or N251-E257 in the N-terminal ARD with the homologous residues of TRPV1, the insertion of valine at position 412 in the pre-S1 domain, the A606V mutation on S5, the inter-subunit disulfide bond F259C-V385C’ in the N-terminal ARD^19,34,38^. Thereby, it is also possible that either manipulation may allosterically disrupt the K614-N647 H-bond in the biggest Grid_11_ or the T411-D519 H-bond in the Grid_4_ to allow the tunable R416-D519 salt bridge in the biggest Grid_17_ to have the lower T_th_ of 32–39 °C for channel opening upon the release of the PC lipid from the active vanilloid site (Figs. 1A, 3A and 5A, Table 1). In support of this proposal, when the Y564A mutation removes the PC lipid from the active vanilloid site, it also has low threshold (37 °C) and Q_10_ (1.21)^22^.

In any way, several smaller grids in the pore domain may be important to stablize the common open state with high heat efficacy. In the pore domain, the first was Grid_6_ with the shortest path from Y594 to T636, I637, F633, L632, Y661, F601 and back to Y594 (Figs. 2A and 5B), and the second was Grid_9_ with the shortest path from D586 to F590, L673 and T680 and then back to D586 (Fig. 2A,C). It has been reported that the T636S mutation decreases the temperature threshold^39^, and the L673F missense mutation was found in a patients with Olmsted syndrome and erythromelalgia^40^. Moreover, the smaller Grid_1_ with the shortest path from S620 to Y622, F654, Q646 and back to S620 in the open state may play a critical role in stabilizing heat efficacy (Fig. 5B). In fact, the mutation N643S, I644S, N647Y, Y661C, or L657I is actually less sensitive to heat^41^. Therefore, it is possible that these mutations may affect the thermostability of these relevant smaller grids in the pore domain.

In contrast, three smallest grids with a zero-residue size in the VSLD may form basic stable backbone anchors to secure mTRPV3 activation or thermal fuses to keep a low systemic thermal instability (Figs. 1A, 2A, 3A and 5B): the first Grid_0_ via the shortest path from F447 to W493, Y451, Y448 and back to F447; the second via the shortest path from Y448 to Y451, N452, W559, F449 and back to Y448; and the third via the shortest path from Y448 to F526, Y564, Y565 and back to Y448.

Some gain-of-function variations in TRPV3 have been reported in patients with hereditary Olmsted syndrome, a rare hyperkeratotic skin channelopathy. They include R416Q/W, W521S, L655P, W692S/G, L694P, G568D/V/C, L673F, G573A/V/S/C. These mutants, in either homomeric or heteromeric form, exhibit differentially elevated basal open probability, increased voltage sensitivity, and cytotoxicity at room temperature 22 °C^42–44^. Of special interest, R416, W521, G568, G573, W692 were located near the PC lipid at the active vanilloid site (Figs. 1A, 2A and 3A). Their mutations may release PC from the active vanilloid site for spontaneous opening. By contrast, L673 was a part of Grid_9_ in the S5/S6 interface and near the lower gate (Figs. 1A, 2A and 3A). Thus, the L673F mutation may perturb the lower gate for spontaneous opening. Further experiments may be required to test how these mutations affect the relevant temperature-dependent noncovalent interactions or the PC binding in the thermorings and thus damage the normal function of TRPV3 in this genetic skin disease. For example, the D519-R416 salt bridge, the H417-R690-E418 and D519-R567 and K614-N647 and S620-Q646 H-bonds, the W521-PC-R567/Q695 bridge, and the Y622-F654-Q646 or Q570-W692-R696-W433-K438 cation/CH-π interactions (Figs. 1A, 2A, 3A and 5B, Tables S1, S2, S3). The better understanding of the structural basis for these variant TRPV3 channels may facilitate further rational design of target-selective drugs.

## Conclusion

In this computational study, a graphical grid thermodynamic model has bridged cryo-electron microscopy-based static conformations with electrophysiological dynamic findings together by using graph theory in atomic details. Once the thermo-rings in the systematic fluidic grid-like mesh network of non-covalent interactions along the PC-dependent minimal gating pathway were tested and identified as key deterministic structural factors or motifs for thermo-gated mTRPV3, three gating states could be in turn established to account for the use-dependent heat sensitization of TRPV3. Accordingly, this grid thermodynamic model can be used precisely to predict the specific thermal stability and activity of cellular biological macromolecules including not only globular proteins but also integral membrane proteins once the high-resolution 3D structural data are available around melting temperature thresholds.

## Methods

### Data mining resources

In this in silico study, the temperature-dependent cryo-EM structures of mTRPV3 in different gating and redox states were analyzed by graph theory to abstract the structural bioinformation for the specific use-dependent temperature thresholds and sensitivity. They included sensitized and open mTRPV3 in the presence of C612-C619 disulfide bond in cNW11 at 42 °C (PDB ID, 7MIN, model resolution = 3.09 Å; 7MIO, model resolution = 3.48 Å, respectively)^24^. Meanwhile, reduced and closed mTRPV3 in MSP2N2 at 4 °C was used as another control for initial heat-sensing (PDB ID, 6LGP, model resolution = 3.31 Å)^23^.

### Standards for non-covalent interactions

In order for the results to be reproduced with a high sensitivity, the structure visualization software, UCSF Chimera, as well as the same rigor and robust standard definition as described and examined previously, was exploited to identify stereo- or regio-selective inter-domain diagonal and intra-domain lateral non-covalent interactions in the 3D structures of mTRPV3 (Tables S1, S2, S3)^28–31^. They included salt-bridges, CH/cation/lone pair/π–π interactions and H-bonds along the PC-dependent minimal gating pathway from D396 to K705 in mTRPV3. Notably, momentary fluctuation-induced changes in the non-bonded interactions during protein dynamics were excluded. In addition, although the hydrophobic effect and residue hydrophobicity are necessary to drive protein folding, their effects on protein stabilization may be rather marginal^45,46^. Finally, only the same group of protein structures obtained in the same model membrane systems was used for comparison unless they were unavailable.

### Preparation of topological grid maps by using graph theory

The filtered non-covalent interactions were geometrically mapped as edges along with marked node arrows to represent the positions of the linked residues in the systematic fluidic mesh network according to the same protocol as previously described and examined^28–31^. All the grids were then covered in this mesh network after their thermoring sizes were constrained as the minimal number of the total free silent side chains of residues or atoms in the bound lipid that did not participate in any non-covalent interction in a grid. The size constraint was completed by using graph theory and the Floyd-Warshall algorithm to calculate the shortest reverse path from one end of a non-covalent interaction to the start in the case that the direct path from the start to the end was zero^47^. For example, in the intra-subunit grid-like biochemical reaction mesh network of Fig. 1A, a direct path length from E610 and N647 was zero because of an H-bond between them. However, there was another shortest reverse path from N647 to K614 and back to E610 via the N647-K614 H-bond and the K614-E610 salt bridge in this grid. Therefore, the grid size was zero. Once all the grid sizes were available, only the uncommon sizes were marked in black, and a grid with an x-residue or atom size was denoted as Grid_x_. When the total number of all noncovalent interactions and grid sizes along the gating pathway were calculated, they were displayed in black and cyan circles beside the mesh network map, respectively, for the calculations of the systematic thermal instability, the structural temperature sensitivity, and the relevant thermodynamic parameters.

### Calculation of the melting temperature threshold of TRPV3

A DNA hairpin thermo-sensor with a 20-base loop and two G-C base pairs in the stem has an empirical start control melting temperature threshold (T_m_) of 34 °C to initiate thermal unfolding of the hairpin loop. When an additional G-C base pair or five additional bases are included in the hairpin, the T_m_ is increased by 10 °C^27^. In a similar way, when a single polypeptide chain in protein carries out rate-limiting thermal unfolding of the thermal rings that range in size from the biggest grid to the smallest grid, the T_m_ of thermal unfolding of the given grid along the chain was calculated by using the following equation as empirically derived from the experimental data^28–31^:

1 $${\text{T}}_{{\text{m}}} (^\circ {\text{C}}) = {34 } + \left( {{\text{n}} - {2}} \right) \times {1}0 + \left( {{2}0 - {\text{S}}_{{{\text{max}}}} } \right) \times {2}$$

where, n is the total number of the grid size-controlled simple H-bonds energetically equivalent to non-covalent interactions in the given grid, and S_max_ is the size of the given grid. In this regard, the more grid’s molar heat capacity would be expected with the decreased grid size or the increased equivalent H-bonds to secure the thermal stability of the protein.

### Calculation of the systemic thermal instability (T_i_)

On the other hand, the T_m_ of the DNA hairpin will be always increased by the more G-C base pairs in the stem or the shorter poly-A loop^27^. Thus, the grid-based systemic thermal instability (T_i_) along the single polypeptide chain was reasonably defined using the following empirical equation as described and examined previously^28–31^:

2 $${\text{T}}_{{\text{i}}} = {\text{S}}/{\text{N}}$$

where, S is the total grid sizes and N is the total non-covalent interactions along the PC-dependent minimal gating pathway of one subunit in a gating state. Usually, the lower T_i_, the less the conformational entropy in the system.

### Calculation of the systematic temperature sensitivity of mTRPV3

For a channel gating transition from a fully closed state to a fully open state driven by the change in the systematic molar enthalpy within a temperature range ∆T, if the chemical potential of a grid is theoretically defined as the maximal potential for equivalent residues in the grid to form the tightest β-hairpin with the smallest loop via paired non-covalent interactions^48^, the grid-based systematic structural thermo-sensitivity (Ω_∆T_) of a single ion channel can be defined and calculated using the following empirical equations as described and examined previously^31^:

3 $$\Omega _{{\Delta {\text{T}}}} = \left[ {\left( {{\text{S}}_{{\text{c}}} - {\text{ S}}_{{\text{o}}} } \right){\text{E}}/2} \right]^{{({\text{Hc}}/{\text{Ho}})}} = \left[ {\left( {{\text{S}}_{{\text{c}}} - {\text{ S}}_{{\text{o}}} } \right){\text{E}}/2} \right]^{{[\left( {{\text{ENc}}} \right)/\left( {{\text{ENo}}} \right){\kern 1pt} ]}} = \left[ {\left( {{\text{S}}_{{\text{c}}} - {\text{ S}}_{{\text{o}}} } \right){\text{E}}/2} \right]^{{({\text{Nc}}/{\text{No}})}}$$

where, in the closed and open states along the same PC-dependent minimal gating pathway of one subunit, H_c_ and H_o_ are the total molar enthalpy included in non-covalent interactions, respectively; S_c_ and S_o_ are the total grid sizes, respectively; N_c_ and N_o_ are the total non-covalent interactions, respectively. E is the molar energy intensity of a non-covalent interaction in a range of 0.5–3 kcal/mol. Usually, E is 1 kcal/mol. Thus, Ω_∆__T_ actually mirrors a heat-evoked change in the total chemical potential of all the grids upon a heat-induced change in the total molar enthalpy included in non-covalent interactions from a closed state to an open state along the same PC-dependent minimal gating pathway of one subunit.

When ∆T = 10 °C, Ω_10_ could be comparable to the functional thermo-sensitivity (Q_10_) of a single ion channel. Q_10_ was defined and calculated using the following equation:

4 $${\text{Q}}_{{{1}0}} = \left( {{\text{X}}_{{2}} /{\text{X}}_{{1}} } \right)^{{{1}0/({\text{T2}} - {\text{T1 }})}}$$

where, X_1_ and X_2_ are open probability (P_o_) values or reaction rates obtained at temperatures T1 and T2 (measured in kelvin), respectively.

### Calculation of the thermodynamic parameters of TRPV3

T_1/2_ was defined as a temperature at which the apparent open probability (P_o_) of mTRPV3 was 0.5. It was calculated using the following equation:

5 $${\text{T}}_{{{1}/{2}}} = {\text{T}}_{{{\text{m}},{\text{c}}}} + \left( {{\text{T}}_{{{\text{m}},{\text{o}}}} - {\text{T}}_{{{\text{m}},{\text{c}}}} } \right)/{2}$$

where, T_m,c_ and T_m,o_ were the calculated melting temperature thresholds of the biggest grids in mTRPV3 in the closed and open states, respectively. At T_1/2_, the change in systematic molar Gibbs free energy (ΔG_1/2_) is zero, and the change in systematic molar enthalpy upon the total broken non-covalent interactions was calculated using the following equation:

6 $$\Delta {\text{H}}_{{1/2}} = 4* \left( {{\text{H}}_{{\text{c}}} - {\text{ H}}_{{\text{o}}} } \right)/2 = 4* \left( {{\text{EN}}_{{\text{c}}} - {\text{EN}}_{{\text{o}}} } \right)/2$$

where, E is usually 1 kcal/mol. Therefore, the change in molar entropy at T_1/2_ could be calculated using the following equation:

7 $$\Delta {\text{S}}_{{1/2}} = \Delta {\text{H}}_{{1/2}} /{\text{T}}_{{1/2}}$$

When the channel was allosterically open at T_m,o_, the change in molar Gibbs free energy along the PC-dependent minimal gating pathway from the closed state at T_m,c_ to the open one at T_m,o_ was calculated using the following equation:

8 $$\Delta {\text{G}} = \left( {{\text{S}}_{{\text{o}}} - {\text{S}}_{{\text{c}}} } \right){\text{E}}/2$$

where, E is usually 1 kcal/mol. Assuming the temperatures of maximal stability (T_s_) were 303.7 and 292 K for the minimal P_o_ of reduced and oxidized channels, respectively, and the enthalpy change at T_s_, ΔH_s_ = – ΔG/2, the concurrent change in molar heat capacity (ΔC_p_) was then calculated from the modified Gibbs–Helmholtz equation to match the maximal experimental molar enthalpy change for channel opening^19^:

9 $$\Delta {\text C}_{{\text{p}}}= (\Delta {\text{H}}_{s}-\Delta {\text{G}}_{1/2})/[({\text T_{\text s}}-{\text T_{1/2}})+{\text T_{1/2}} * \ln ({\text T_{1/2}}/{\text T_{\text s}})]$$

### Supplementary Information

Supplementary Information.

## Data Availability

All data generated or analysed during this study are included in this published article and Supplementary Information.

## Abbreviations

ARD: Ankyrin repeat domain
cryo-EM: Cryo-electron microscopy
T_m_: Melting temperature threshold
MD: Molecular dynamics
PC: Phosphatidylcholine
PI: Phosphatidylinositol
T_th_: Temperature threshold
TMD: Transmembrane domain
TRP: Transient receptor potential
TRPA: TRP ankyrin
TRPC: TRP canonical
TRPVi: TRP vanilloid i
TRPV3: TRP vanilloid-3
hTRPV1: Human TRPV1
rTRPV1: Rat TRPV1
mTRPV3: Mouse TRPV3
TRPMi: TRP melastatin i
TRPM8: TRP melastatin 8
VSLD: Voltage-sensor-like domain
