# Author Correction: Thermoring basis for the TRPV3 bio-thermometer

**DOI:** 10.1038/s41598-024-55395-w

**Published:** 2024-02-28

**Authors:** Guangyu Wang

**Affiliations:** 1 Department of Physiology and Membrane Biology, University of California School of Medicine, Davis, CA 95616 USA; 2 Department of Drug Research and Development, Institute of Biophysical Medico-Chemistry, Reno, NV 89523 USA

Correction to: *Scientific Reports* 10.1038/s41598-023-47100-0, published online 07 December 2023

The original version of this Article contained errors.

In the original version of this Article, the calculated heat capacity of homotetrameric TRPV3 was incorrectly calculated.

As a result, Table 1

**Table:** 

Construct	Wild-type mTRPV3					
∆H_1/2_, kcal/mol	**1**	** − 0.5**			**2**	**2.5**
∆S_1/2_, cal/mol-K	**3.03**	** − 1.52**			**6.18**	**7.73**
∆Cp, kcal/mol-K	**604**	**670**			**14.4**	**35.1**
Systemic thermal instability (T_i_)	**1.88**	**1.95**	**1.20**	**1.25**	**1.21**	**1.33**
Calculated Ω_10, min_ at E_min_ = 0.5 kcal/mol	10.1	9.78			1.27	3.28
Calculated Ω_10, mean_ at E_mean_ = 1.0 kcal/mol	**20.8**	**19.3**			**2.69**	**6.92**
Calculated Ω_10, max_ at E_max_ = 3.0 kcal/mol	65.2	56.6			8.81	22.7
Measured Q_10,_	**20.6**				**2.32**	
Ref. for measured T_th_ or Q_10_	^19^		^19,33^		^19,24^	

now reads,

**Table:** 

Construct	Wild-type mTRPV3					
∆H_1/2_, kcal/mol	**4**	** − 2**			**8**	**10**
∆S_1/2_, cal/mol-K	**12.1**	** − 6.07**			**24.7**	**30.9**
∆Cp, kcal/mol-K	**8.68**	**9.71**			**0.762**	**1.83**
Systemic thermal instability (T_i_)	**1.88**	**1.95**	**1.20**	**1.25**	**1.21**	**1.33**
Calculated Ω_10, min_ at E_min_ = 0.5 kcal/mol	10.1	9.78			1.27	3.28
Calculated Ω_10, mean_ at E_mean_ = 1.0 kcal/mol	**20.8**	**19.3**			**2.69**	**6.92**
Calculated Ω_10, max_ at E_max_ = 3.0 kcal/mol	65.2	56.6			8.81	22.7
Measured Q_10,_	**20.6**				**2.32**	
Ref. for measured T_th_ or Q_10_	^19^		^19,34^		^19,24^	

In addition, in the Results section

“Assuming the channel open probability (P_o_) was 0.5 at T_1/2_ of 50.5 °C (323.5 K), which was close to the experimental value^19^, the change in molar enthalpy at 323.5 K (ΔH_1/2_) would be 2 kcal/mol (Table 1). Thus, the change in molar entropy at 323.5 K (ΔS_1/2_) was 6.18 cal/mol-K (Table 1). Assuming mTRPV3 was fully open at 61 °C, the change in molar Gibbs free energy (ΔG) would be − 2.5 kcal/mol (Table 1). Based on the Gibbs–Helmholtz equation, the change in molar heat capacity (ΔC_p_) was about 14.4 kcal/mol-K for one subunit (Table 1). When the gating pathway was extended beyond the PC-dependent minimal gating pathway, the ΔC_p_ value increased to 35.1 kcal/mol-K (Table 1).

In contrast, for channel gating from the closed and reduced state at 52 °C to the open and oxidized one until the maximal activity temperature around 61 °C, the total noncovalent interactions and grid sizes along the PC-dependent minimal gating pathway decreased from 51 and 96 to 49 and 59, respectively (Table 1). If P_o_ was 0.5 at T_1/2_ of 56.5 °C (329.5 K), which was still near the experimental value^19^, ΔH_1/2_ and ΔS_1/2_ at 329.5 K would be 1 kcal/mol and 3.03 cal/mol-K, respectively (Table 1). If mTRPV3 completely opened at 61 °C, ΔG would be − 18.5 kcal/mol (Table 1). Using the same Gibbs–Helmholtz equation, ΔC_p_ was calculated as large as 604 kcal/mol-K for one subunit (Table 1). The value increased to 670 kcal/mol-K along with the extended gating pathway from F377 to W742 (Table 1).”

now reads,

“Assuming the apparent channel open probability (P_o_) was 0.5 at T_1/2_ of 50.5 °C (323.5 K), which was close to the experimental value^19^, the change in molar enthalpy at 323.5 K (ΔH_1/2_) upon the total broken non-covalent interactions would be 8 kcal/mol (Table 1). Thus, the change in molar entropy at 323.5 K (ΔS_1/2_) was 24.7 cal/mol-K (Table 1). Assuming mTRPV3 was allosterically open at 61 °C, the change in molar Gibbs free energy (ΔG) would be − 2.5 kcal/mol (Table 1). Based on the modified Gibbs–Helmholtz equation, the change in molar heat capacity (ΔC_p_) was about 0.762 kcal/mol-K for one channel (Table 1). When the gating pathway was extended beyond the PC-dependent minimal gating pathway, the ΔC_p_ value increased to 1.83 kcal/mol-K (Table 1).

In contrast, for channel gating from the closed and reduced state at 52 °C to the open and oxidized one until the maximal activity temperature around 61 °C, the total noncovalent interactions and grid sizes along the PC-dependent minimal gating pathway of one subunit decreased from 51 and 96 to 49 and 59, respectively (Table 1). If the apparent P_o_ was 0.5 at T_1/2_ of 56.5 °C (329.5 K), which was still near the experimental value^19^, ΔH_1/2_ and ΔS_1/2_ at 329.5 K upon the total broken non-covalent interactions would be 4 kcal/mol and 12.1 cal/mol-K, respectively (Table 1). If mTRPV3 allosterically opened at 61 °C, ΔG would be − 18.5 kcal/mol (Table 1). Using the same Gibbs–Helmholtz equation, ΔC_p_ was calculated as large as 8.68 kcal/mol-K for one channel (Table 1). The value increased to 9.61 kcal/mol-K along with the extended gating pathway from F377 to W742 (Table 1).”

Furthermore, in the Discussion section

“It should be noteworthy that even if the change in molar enthalpy (ΔH) of one subunit was as small as 2 kcal/mol from a closed and reduced state to an oxidized and open one, the huge change in molar heat capacity (ΔC_p_) of 604 kcal/mol-K was still enough to absorb more heat as the configurational heat capacity (love) while keeping a small change in molar entropy at T_1/2_ (ΔS_1/2_, 3.03 cal/mol). This observation was in agreement with the decrease in the conformational entropy as reflected by the decrease of the systematic thermal instability from 1.88 to 1.20 to stabilize the open state (Table 1). In sharp contrast, when mTRPV3 was oxidized, ΔC_p_ dramatically declined from 604 kcal/mol-K to 14.4 kcal/mol-K along with the decrease in the mean Ω_10_ value from 20.8 to 2.69 and the decline in Q_10_ from 20.6 to 2.32 (Table 1).”

now reads

“It should be noteworthy that even if the change in molar enthalpy (ΔH) of one channel upon the total broken non-covalent interactions was as small as 8 kcal/mol from a closed and reduced state to an oxidized and open one, the huge change in molar heat capacity (ΔC_p_) of 8.68 kcal/mol-K was still enough to absorb more heat as the configurational heat capacity (love) while keeping a small change in molar entropy at T_1/2_ (ΔS_1/2_, 12.1 cal/mol). This observation was in agreement with the decrease in the entropy of compact conformations as reflected by the decrease of the systematic thermal instability from 1.88 to 1.20 to stabilize the apparent open state (Table 1). In sharp contrast, when mTRPV3 was oxidized, ΔC_p_ dramatically declined from 8.68 kcal/mol-K to 0.762 kcal/mol-K along with the decrease in the mean Ω_10_ value from 20.8 to 2.69 and the decline in Q_10_ from 20.6 to 2.32 (Table 1).”

Finally, in the Methods section

“T_1/2_ was defined as a temperature at which the open probability (P_o_) of mTRPV3 was 0.5. It was calculated using the following equation:

$$ {\text{T}}_{{{1}/{2}}} = {\text{ T}}_{{{\text{m}},{\text{c}}}} + {\text{ (T}}_{{{\text{m}},{\text{o}}}} {-}{\text{ T}}_{{{\text{m}},{\text{c}}}} {)}/{2} $$

where, T_m,c_ and T_m,o_ were the calculated melting temperature thresholds of the biggest grids in mTRPV3 in the closed and open states, respectively. At T_1/2_, the change in systematic molar Gibbs free energy (ΔG) is zero. The change in systematic molar enthalpy was calculated using the following equation:

$$ \Delta {\text{H}}_{{{1}/{2}}} = {\text{ (H}}_{{\text{c}}} {-}{\text{ H}}_{{\text{o}}} {)}/{2 } = {\text{ (EN}}_{{\text{c}}} {-}{\text{ EN}}_{{\text{o}}} {)}/{2} $$

where, E is usually 1 kcal/mol. Therefore, the change in molar entropy at T_1/2_ could be calculated using the following equation:

$$ \Delta {\text{S}}_{{{1}/{2}}} = \, \Delta {\text{H}}_{{{1}/{2}}} /{\text{T}}_{{{1}/{2}}} $$

When the channel was fully open at T_m, o_, the change in molar Gibbs free energy along the PC-dependent minimal gating pathway from the closed state at T_m,c_ to the open one at T_m,o_ was calculated using the following equation:

$$ \Delta {\text{G}}_{{{\text{Tm}},{\text{o}}}} = {\text{ (S}}_{{\text{o}}} - {\text{ S}}_{{\text{c}}} {\text{)E}}/{2} $$

where, E is usually 1 kcal/mol. So, the concurrent change in molar heat capacity (ΔCp) was calculated from the Gibbs–Helmholtz equation:

$$ \Delta {\text{C}}_{{\text{p}}} =  \{ \Delta {\text{H}}_{{{1}/{2}}} [{1 } - {\text{ (T}}_{{{\text{m}},{\text{o}}}} /{\text{T}}_{{{1}/{2}}} {)}] \, - \, \Delta {\text{G}}_{{{\text{Tm}},{\text{o}}}} \}/[({\text{T}}_{{{1}/{2}}} - {\text{ T}}_{{{\text{m}},{\text{o}}}} ) \, + {\text{ T}}_{{{\text{m}},{\text{o}}}} *{\text{ ln(T}}_{{{\text{m}},{\text{o}}}} /{\text{T}}_{{{1}/{2}}} {)}] {\text '}{\text '}$$

now reads,

“T_1/2_ was defined as a temperature at which the apparent open probability (P_o_) of mTRPV3 was 0.5. It was calculated using the following equation:

$$ {\text{T}}_{{{1}/{2}}} = {\text{ T}}_{{{\text{m}},{\text{c}}}} + {\text{ (T}}_{{{\text{m}},{\text{o}}}} {-}{\text{ T}}_{{{\text{m}},{\text{c}}}} {)}/{2} $$

where, T_m,c_ and T_m,o_ were the calculated melting temperature thresholds of the biggest grids in mTRPV3 in the closed and open states, respectively. At T_1/2_, the change in systematic molar Gibbs free energy (ΔG_1/2_) is zero, and the in systematic molar enthalpy upon the total broken non-covalent interactions was calculated using the following equation:

$$ \Delta {\text{H}}_{{{1}/{2}}} = { 4}*({\text{H}}_{{\text{c}}} {-}{\text{ H}}_{{\text{o}}} )/{2 } = { 4}*({\text{EN}}_{{\text{c}}} {-}{\text{ EN}}_{{\text{o}}} )/{2} $$

where, E is usually 1 kcal/mol. Therefore, the change in molar entropy at T_1/2_ could be calculated using the following equation:

$$ \Delta {\text{S}}_{{{1}/{2}}} = \, \Delta {\text{H}}_{{{1}/{2}}} /{\text{T}}_{{{1}/{2}}} $$

When the channel was allosterically open at T_m, o_, the change in molar Gibbs free energy along the PC-dependent minimal gating pathway from the closed state at T_m,c_ to the open one at T_m,o_ was calculated using the following equation:

$$ \Delta {\text{G}} = {\text{ (S}}_{{\text{o}}} - {\text{ S}}_{{\text{c}}} {\text{)E}}/{2} $$

where, E is usually 1 kcal/mol. Assuming the temperatures of maximal stability (T_s_) were 303.7 and 292 K for the minimal P_o_ of reduced and oxidized channels, respectively, and the enthalpy change at T_s_, ΔH_s_ = – ΔG/2; the concurrent change in molar heat capacity (ΔC_p_) was then calculated from the modified Gibbs–Helmholtz equation to match the maximal experimental molar enthalpy change for channel opening^19^:

$$ \Delta {\text{C}}_{{\text{p}}} = { (}\Delta {\text{H}}_{{\text{s}}} {-} \, \Delta {\text{G}}_{{{1}/{2}}} {)}/{ [}({\text{T}}_{{\text{s}}} {-}{\text{ T}}_{{{1}/{2}}} ) \, + {\text{ T}}_{{{1}/{2}}} + {\text{ T}}_{{{1}/{2}}} *{\text{ ln(T}}_{{{1}/{2}}} /{\text{T}}_{{\text{s}}} {)]}  {\text '}{\text '}$$

The original Article has been corrected.

