# Supplementary material for: Thermoring basis for the TRPV3 bio-thermometer
Source: Sci Rep. 2023 Dec 7;13:21594. doi: 10.1038/s41598-023-47100-0 (PMC10703924; doi:10.1038/s41598-023-47100-0)
Supplement: Supplementary file 1 — Supplementary Information. [file 41598_2023_47100_MOESM1_ESM.pdf]

Supplementary Information for

Thermoring basis for the TRPV3 bio-thermometer

Running title: Digit codes in thermo-gated TRPV3

Guangyu Wang 1, 2*

[1]Department of Physiology and Membrane Biology, University of California School of Medicine, Davis, CA 95616, USA

[2]Department of Drug Research and Development, Institute of Biophysical Medico-chemistry, Reno, NV 89523, USA

* Correspondence: gary.wang10@gmail.com

This supplementary material includes:

Tables S1-S3.

Table S1. Noncovalent interactions along the PC-dependent minimal gating pathway from D396 to K705 in each subunit of closed PC-bound oxidized mTRPV3 at 42 °C after heat sensitization

**Noncovalent interaction**	**Cut-off distance**	**Linked residues**
Salt bridge	3.2-4 Å	D396-K432-E704, R416-D519, E610-K614, **R698-E702**
H-bond	<3.9 Å donor-H-acceptor <60°	T397-E704, T397-K432, Y448-Q529, **T456-W559**, **K500-E501,** R567-T699, **D586-T680**, **Y594-T636**, E610-N647-K614, T636-Y661, E682-K686, **E689-R693,** Q695-PC
π–π interaction	2.65–6.5 Å	**W433-F441-Y565, F445-Y565, F447-W493-F489**, **Y448-Y451**, **Y448-F526**, **Y448-Y565**, **F449-W559**, **Y451-W493**, **Y460-Y461**, **H471-Y540, H471-Y547**, **W521-F522**, F522-F526, **F522-Y564, F526-Y564**, **Y540-Y547**, **F542-Y544**, **Y564-Y565**, **F601-Y661**, **Y622-F654**
cation–π interaction	<6.0 Å	**R696-W433**
CH_3/CH–π interaction	2.65-3.01 Å	**W433-K438**-Y439, **F449-I453, N452-W559,** W521-PC, **F527-V531, F590-L673**, F597-F601, F597-L664
Lone pair–π interaction	3-3.7 Å	**F656-T660, T665-Y661**

Note: Bold interactions were conserved in both closed and open states.

Table S2. Noncovalent interactions along the PC-dependent minimal gating pathway from D396 to K705 in each subunit of open PC-free oxidized mTRPV3 at 42 °C after heat sensitization

Noncovalent interaction	Cut-off distance	Linked residues
Salt bridge	3.2-4 Å	K500-E501, E689-R693, **R698-E702**
H-bond	<3.9 Å donor-H-acceptor <60°	T411-R416, H417-R690-E418, E423-T427, K432-E704, **T456-W559,** D519-R567, **D586-T680, Y594-T636,** S520-Q646, E631-K634
π–π interaction	2.65–6.5 Å	**W433-F441-Y565-F445**, **F447-W493-F489**, **Y448-Y451, Y448-F526**, **Y448-Y565**, **F449-W559**, **Y451-W493**, **Y460-Y461**, **H471-Y540**, **H471-Y547**, **W521-F522**, **F522-Y564**, **F526-Y564**, **Y540-Y547**, **F542-Y544**, **Y564-Y565**, **F601-Y661**, **Y622-F654**
cation–π interaction	<6.0 Å	**R696-W433**
CH_3/CH–π interaction	2.65-3.01 Å	**W433-K438, F449-I453**, **N452-W559**, F527-V531, **Q570-W692**, **F590-L673**, F625-V629, L632-Y661, F633-I637, **Q646-F654**, W692-R696
Lone pair–π interaction	3-3.7 Å	**F656-T660, T665-Y661**

Note: Bold interactions were conserved in both closed and open states.

Table S3. Noncovalent interactions along the PC-dependent minimal gating pathway from D396 to K705 in each subunit of closed PC-bound reduced mTRPV3 at 4 °C without heat sensitization

**Noncovalent interaction**	**Cut-off distance**	**Linked residues**
Salt bridge	3.2-4 Å	D396-K432-E704, R416-D519-R698, R567-PC
H-bond	<3.9 Å donor-H-acceptor <60°	T397-E704, T411-D519, Y448-Q529-Y451, Q514-S518, T566-S576, Q570-E689, **D586-T680**, **Y594-T636**, Y594-Y661, K614-N647, T636-Y661, E689-R693
π–π interaction	2.65–6.5 Å	**W433-F441-Y565, F445-Y565, F447-W493-F489, Y448-Y451**, **Y448-F526**, **Y448-Y565**, **F449-W559, Y451-W493**, **Y460-Y461**, **W521-F522**, W521-F524, **F522-Y564, F526-Y564**, **Y540-Y547**, **Y564-Y565**, **F601-Y661**, **Y622-F654**
cation–π interaction	<6.0 Å	**R696-W433**
CH_3/CH–π interaction	2.65-3.01 Å	**W433-K438-**Y439**, N452-W559,** W521-PC, F524-V528, **F527-V531,** F590-Y594, **F590-L673**, **F625-V629, F633-I637,** T649-Y650
Lone pair–π interaction	3-3.7 Å	**F656-T660, T665-Y661**

Note: Bold interactions were conserved in both closed and open states.
